# Response to Garcia and Wanner “gender inequality and food security: lessons for the gender-responsive work of the international food policy research institute and the Bill and Melinda Gates Foundation” *Food Security* (2017) 9:1091–1103

**DOI:** 10.1007/s12571-018-0766-7

**Published:** 2018-01-26

**Authors:** Ruth Meinzen-Dick, Agnes Quisumbing

**Affiliations:** 1 International Food Policy Research Institute, Washington, DC, USA

## Abstract

The paper by Garcia and Wanner reviews examples of gender-responsive research and programming from the International Food Policy Research Institute (IFPRI) and the Bill & Melinda Gates Foundation, to discuss those gender-responsive approaches which are most successful for achieving women’s empowerment in agricultural development. It notes accomplishments and points out some shortcomings for both organizations to address. Although the objectives of the paper are worthwhile, we note important problems regarding methodology used, factual errors, and misrepresentation.

## 1 Comments on methodology

The paper’s methodology is flawed in two important ways.

First, it is based on only five key informant interviews (including only one each from IFPRI and the Bill and Melinda Gates Foundation, which are the focus of the study). The justification on p. 1093 for the limited reach stated that “The seemingly small number of 5 key informant interviewees used for our analysis can be explained by a lack of willingness of IFPRI and BMGF to be interviewed” is misleading, at best. To our knowledge, only two researchers at IFPRI were contacted. One was interviewed; the other was on leave when the request arrived, and responded upon return from leave, only to be informed that the interview was not needed.

Second, the methodology relies on a very limited reading of both organizations’ documents (based on the cited literature). For example, the only citation for the Women’s Empowerment in Agriculture Index (WEAI) is a powerpoint presentation. Had the authors read some of the other materials on this (which had been recommended to the authors or are available on the WEAI Resource Center https://www.ifpri.org/topic/weai-resource-center), they could have avoided some of the factual errors noted below.

Comments on factual errors and misrepresentation:

The factual errors and misrepresentation in this paper can be classified into three types: (1) mistaking research for implementation; (2) errors regarding the Women’s Empowerment in Agriculture Index; and (3) conflating gender research and gender staffing.

(1) Mistaking research for implementation

IFPRI is a research institution. It does not implement nor fund agricultural development projects.Yet, on p. 1095, the authors state: “Gender-responsive projects run and co-created by IFPRI are currently focused on the provision of agricultural assets.” This seems to be based on a misinterpretation of the Gender, Agriculture and Assets Project (GAAP), in which IFPRI conducted impact evaluations of eight project interventions implemented by various development agencies to discern the impacts of these interventions on gender-related outcomes of interest. IFPRI did not recommend the types of strategies implemented by the projects in GAAP. Contrary to Garcia and Wanner’s statement (p. 1095) that “The GAAP report stipulates that the approaches used by the project implementers utilizing agricultural assets are in line with IFPRI’s goals and agendas concerning gender and agricultural development (Johnson et al. 2015,2016)” that source states that the projects being evaluated “took diverse approaches to gender—ranging from gender blind to gender transformative” (Johnson et al. 2015:1). The intention was to learn how different types of interventions addressed the gender asset gap, rather than to endorse any of these strategies ex ante.^1^

Instead, projects were already designed and underway when they applied to join the GAAP initiative. Thus, the statement on p. 1100 is also erroneous: “While IFPRI did not implement the GAAP assisted projects directly, it explicitly promotes the provision of assets to implementors of agricultural development projects and does not provide solutions or measures for avoiding the serious consequences encountered (Johnson et al. 2015). It is imperative that IFPRI addresses these issues thoroughly and provides advice accordingly.” First, the projects, not IFPRI, determined their asset-related strategies. Second, the lack of attention to unintended consequences is a misreading of IFPRI’s work on gender. In numerous publications, such as Haddad et al. (1997), Quisumbing (2003), the Johnson et al. (2015) synthesis paper and the source publications from GAAP on which that synthesis was based,^2^ IFPRI researchers have written extensively about the unintended consequences of agricultural development projects, including those that transfer assets.

Possibly the most serious misrepresentation is the implication that IFPRI projects increase domestic violence: (p. 1101) “IFPRI experienced increases in domestic violence and added time burdens for women following the provisioning of agricultural assets in GAAP project initiative (Johnson et al. 2015) …The analysis of IFPRI’s gender-responsive projects suggests that these risks are not outlined by the institute in their public documents and strategies.” As mentioned above, IFPRI does not implement development projects, but evaluates them. Without rigorous, systematic evaluations such as those implemented under GAAP, we would not know about the unintended consequences. In fact, other IFPRI work has focused on women’s time burdens (see the systematic review by Johnston et al. 2015), and a body of literature (including IFPRI research) on domestic violence and transfers, in the context of social protection programs has demonstrated that transfers tend to actually decrease domestic violence, on average (Angelucci, 2008 and Bobonis et al., 2013 in Mexico; Hidrobo et al., 2016 in Ecuador; Perova & Vakis, 2013 in Peru; Haushofer & Shapiro, 2016 in Kenya; Roy et al., 2017 in Bangladesh). Some of IFPRI’s projects on gender involve long-term tracking efforts to ascertain long-term impacts on domestic violence, women’s and children’s nutritional status, etc., although, as the authors correctly point out, long-term follow-ups are constrained by funding. Within our research programs, the IFPRI Institutional Review Board carefully examines each study for potential negative effects on participants.

Finally, among the eight projects that were part of the GAAP portfolio, only one mentioned an increase in domestic violence incidents, and this was not even an asset transfer project but instead a value chain project. Upon finding out about these incidents through the IFPRI impact evaluation, the project implemented a community-based intervention to raise awareness among men and engage them in helping to address the problem. This is mentioned in the Johnson et al. (2015) paper, but was omitted by Garcia and Wanner.

(2) Factual errors regarding representation of the Women’s Empowerment in Agriculture Index (WEAI)

Garcia and Wanner (2017) make many errors in the description of the Women’s Empowerment in Agriculture Index (WEAI). The development of WEAI was not funded by the Bill & Melinda Gates Foundation, as the authors suggest (p. 1097), but by USAID. The description of the WEAI is also wrong: as a survey-based index based on interviews of the primary male and female decision makers in agricultural households, it does not “utilize points and indicators” (p. 1097) to measure empowerment. The WEAI was developed jointly by IFPRI, the Oxford Poverty and Human Development Initiative and USAID, and its methodological roots are grounded in the Alkire-Foster family of indices, which is explained in Alkire et al. (2013)—a reference that was provided to the authors, but apparently not used.

We were surprised to find WEAI scores in the table listing the GAAP projects (Table 2 in the Garcia and Wanner paper). The WEAI did not exist when GAAP started, so the WEAI scores would not have come from the projects themselves, nor from Johnson et al. (2015), which is cited as the source for the table. If these scores were extrapolated from the population-based surveys, they would not be relevant for the households in the GAAP studies, because these samples, being drawn from impact evaluations, would not have been statistically representative of the populations from which the Feed the Future Initiative’s Population-Based Surveys (PBS) were drawn. So, the authors’ statement that “Our analysis …suggests an overall low women’s empowerment impact when utilizing the WEAI (Table 2)” has no empirical (or other) basis.

Further, Garcia and Wanner (2017:1097) note that “A major flaw in its [WEAI] design is that it does not include indicators for measuring increased nutrition which is a significant area of concern in food security”. As explained in Alkire et al. (2013:82), the WEAI indicators “deliberately focused only on empowerment in agriculture. The precision of the measure creates a strength for analysis: We can easily scrutinize how empowerment in women’s specific agricultural roles relates to other aspects of their resources and outcomes (Kabeer 1999) as well as their empowerment in other areas.” Indeed, the relationship between women’s empowerment in agriculture and nutrition outcomes has been studied by Sraboni et al. (2014) for Bangladesh, Malapit and Quisumbing (2015) for Ghana, and Malapit et al. (2015) for Nepal–all available on the WEAI Resource Center.

(3) Conflating gender research and gender staffing

Sections 6.3–6.5 in Garcia and Wanner (2017) mix up the distinct areas of gender research and the gender composition of research staff. Section 6.3 appears to be advocating for inclusion of more women in research, whereas section 6.5 advocates for inclusion of men. Section 6.4 (p. 1099) concludes that “BMGF is more advanced in this respect than IFPRI which does not seem to have internal mechanisms to change gender structures within its organisation. While IFPRI’s gender research staff are primarily women, it should increase the emphasis it places on gender equality through internal gender initiatives.” But the authors did not request or review IFPRI’s gender staffing policies; there is no basis for this statement, and because gender is a cross-cutting theme of IFPRI’s research, many men also do research on gender in other work programs.

## 2 Comments on conclusions and implications

While we agree with the importance of studying the long-term impacts of interventions, the statement on p.1101 is misinformed: “Additionally, the long-term impacts of these projects are unclear and can only be determined through ongoing analyses of women’s empowerment in the areas focused on by research programs. *This area requires major improvement in IFPRI’s gender work as there is no indication of intentions for long-term impact assessment of IFPRI’s research projects.”* (italics ours).

This is a clear misreading of IFPRI’s work, probably directly due to the poor methodology chosen for the paper. There have been ex post evaluations of IFPRI’s gender research (e.g. Jackson 2005; Baden et al. 2017) and IFPRI has a track record of research on long-term impacts. One such example is the Pathways from Poverty Research program, which involved longitudinal surveys that resurveyed households 10–25 years after an intervention took place. This research program analyzed data from households and individuals to examine long-term impacts on gender dynamics. A study in Bangladesh, for example, found that vegetable technology programs targeted to women through women’s groups, had better long-term impacts on women’s and girls’ nutritional status compared to fishpond programs that were targeted to the household, and by default, to the male in that household (Kumar and Quisumbing 2011).

Similarly, IFPRI’s work in Ethiopia, which surveyed the same households before and after the reform of the Family Code and the community-based land registration program, found that gender norms had shifted towards gender equality after these mutually-reinforcing reforms were implemented (Kumar and Quisumbing 2015). The reform in the Family Code improved divorce settlements for women; this is important because women who perceived that divorce settlements were less favorable towards them tended to invest less in their daughters’ schooling—showing that these reforms indeed have long-term and even intergenerational impacts (Kumar and Quisumbing 2012).

Finally, the Transfer Modality Research Initiative in Bangladesh (TMRI) followed up recipients of food and cash transfers, with or without behavior change communication, and found that the combination of transfers and behavior change communication reduced the prevalence of domestic violence 6–10 months after the intervention had ended (Roy et al. 2017). There are plans for a longer-term follow-up of TMRI, 4 years after the intervention. It is true that funding constraints do not permit researchers to conduct follow-up studies for many projects. But many IFPRI surveys are designed to make follow-ups possible, and if funding permits, the follow-ups are conducted. IFPRI has also consistently raised the issue of lack of funding for ex-post follow-ups with donors.

We agree with the recommendation for thorough, preliminary, context specific research, but take exception to the implication of the statement on p. 1101 that “IFPRI experienced increases in domestic violence … ” IFPRI, as an institution, has not experienced domestic violence and development projects that applied for impact assessment to GAAP did not experience domestic violence resulting from the impact assessment. Rather, IFPRI’s impact assessment *identified* increased domestic violence (albeit only in a single project), which was then addressed by the implementer.

As to the recommendation (p. 1101) that “IFPRI should put more resources into encouraging preliminary research in communities concerning socially perceived gender roles and potential time burdens resulting from the provision of assets”, IFPRI conducts research using quantitative and qualitative methods within both phases of GAAP and the rest of its gender work. The qualitative work, in particular, aims to get at local definitions of gender roles, and how these may affect the take-up as well as the impact of development interventions. IFPRI does not undertake this work to prepare for project implementation because, as mentioned above, IFPRI is a research institution, not an implementing organization. IFPRI may work with implementing organizations to design pilot interventions, which are then systematically evaluated; in this case, IFPRI researchers will undertake intensive formative research in the communities.

We share with the authors a concern for reducing gender inequality, and feel that high-quality research can contribute to this goal. Unfortunately, Garcia and Wanner’s paper does not represent high quality research, with inadequate methods leading to flawed results and conclusions.

## Footnotes

1 It should be noted that not all the projects in the GAAP portfolio were asset transfer programs; some aimed to increase the returns to assets (such as value chain programs).

2 See http://gaap.ifpri.info/findings/ for a full list of peer-reviewed journal articles and other publications from GAAP.
